# Optimizing Safety in Robotic-Assisted Right Hepatectomy With Techniques for Complete Vascular Control of the Left and Middle Hepatic Veins: A Case Report

**DOI:** 10.7759/cureus.112644

**Published:** 2026-07-14

**Authors:** Justin M Bader, David Su, Sean Liu, Kevin Billingsley

**Affiliations:** 1 Cancer Research, National Cancer Institute, National Institutes of Health, Bethesda, USA; 2 Surgery, Yale New Haven Hospital, New Haven, USA; 3 Department of Surgery, Yale University, New Haven, USA; 4 Surgical Oncology, Yale New Haven Hospital, New Haven, USA

**Keywords:** complete vascular control, complex hepatic surgery, innovative operative techniques, operative safety, robotic-assisted surgery

## Abstract

Robotic right hepatectomy remains technically challenging, particularly due to the risk of bleeding from the left and middle hepatic veins during parenchymal transection. We present the case of a 71-year-old woman with colorectal adenocarcinoma and an isolated segment 7 hepatic metastasis involving the right hepatic vein who underwent robotic-assisted right hepatectomy after neoadjuvant chemotherapy. Key operative maneuvers were employed to ensure safe vascular control, including extrahepatic control of the left and middle hepatic veins, intraoperative ultrasound guidance for vascular mapping, indocyanine green (ICG) assessment of hepatic perfusion, and intermittent Pringle maneuver application during parenchymal transection. The estimated blood loss was 100 mL, with no transfusion requirement or bile leak. This case demonstrates that complete inflow and outflow control during right hepatectomy is feasible with the robotic platform and may improve operative safety in these patients.

## Introduction

Robotic liver surgery has expanded rapidly over the past decade, offering improved visualization, instrument articulation, and access to difficult anatomic planes compared with conventional laparoscopic and open approaches [1-4]. Despite these advantages, robotic right hepatectomy remains a technically demanding operation due to the complexity of dissection, hepatic inflow and outflow control, and parenchymal transection near major vascular structures [5-7]. In particular, bleeding from the middle hepatic vein or its branches during parenchymal transection can be difficult to control and remains a challenge even for the most experienced surgeons [8,9]. If not adequately controlled, patients may require blood transfusions, modification of the planned resection, or sometimes, conversion to an open approach [8,9]. Furthermore, in patients with tumor involvement of the right hepatic vein or posterior right liver, these challenges are amplified by the need to achieve an oncologic resection while preserving adequate venous drainage of the future liver remnant [10,11].

Early vascular control is a key principle in complex hepatic surgery, yet strategies for complete control of the left and middle hepatic veins during robotic right hepatectomy have not been widely described. Although the primary venous bleeding risk during right hepatectomy arises along the transection plane from the middle hepatic vein and its tributaries, the left and middle hepatic veins often share a close extrahepatic confluence, making control of the left-middle hepatic venous trunk a practical strategy for venous outflow control. We present the case of a robotic-assisted right hepatectomy with innovative intraoperative maneuvers, including extrahepatic vascular control of the left and middle hepatic veins, intraoperative ultrasound guidance for vascular mapping, indocyanine green (ICG) assessment of hepatic perfusion, and use of intermittent Pringle maneuver during liver transection. These techniques highlight a reproducible approach to gain early and complete vascular control, which could potentially improve operative safety during complex robotic liver resection.

## Case presentation

A 71-year-old woman with hypertension, chronic kidney disease, COPD, atrial fibrillation on apixaban, congestive heart failure, and no prior abdominal surgeries initially presented in 2022 after an episode of complicated appendicitis managed non-operatively with antibiotics. During that admission, she was found to have rectal bleeding and subsequently underwent colonoscopy, which demonstrated a cecal mass with biopsy-confirmed invasive adenocarcinoma. She underwent laparoscopic-assisted right hemicolectomy in August 2022, with final pathology demonstrating a margin-negative, mismatch repair-proficient adenocarcinoma with 30 negative lymph nodes. She recovered well postoperatively and was followed with surveillance imaging.

**Figure 1:**
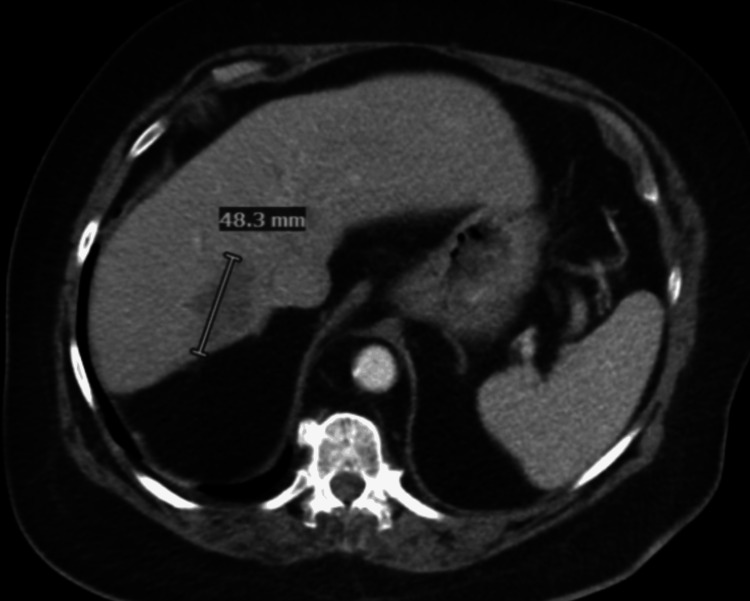
Preoperative abdominal pelvis CT scan with IV contrast visualizing a hepatic lesion in Segment 7.

Surveillance imaging a year later in 2023 demonstrated a small, 1.4 cm lesion in hepatic segment 7, which was initially indeterminate and managed with annual contrast-enhanced CT scan surveillance. The hepatic lesion demonstrated interval enlargement on imaging to 2.3 cm in 2024 and 4.8 cm in 2025 (Figure 1) with concern for new right hepatic vein involvement. Biopsy of the hepatic lesion confirmed metastatic adenocarcinoma consistent with a colorectal primary. There was no evidence of additional intrahepatic disease or distant metastases. Given the solitary hepatic metastasis and patient comorbidities, a minimally invasive approach using the robotic platform was chosen. She received neoadjuvant systemic chemotherapy with five cycles of FOLFOX over two months, with radiographic response of the segment 7 lesion and no suspected chemotherapy-related liver injury. However, due to persistent involvement of the right hepatic vein, a right hepatectomy was required for complete resection.

**Figure 2:**
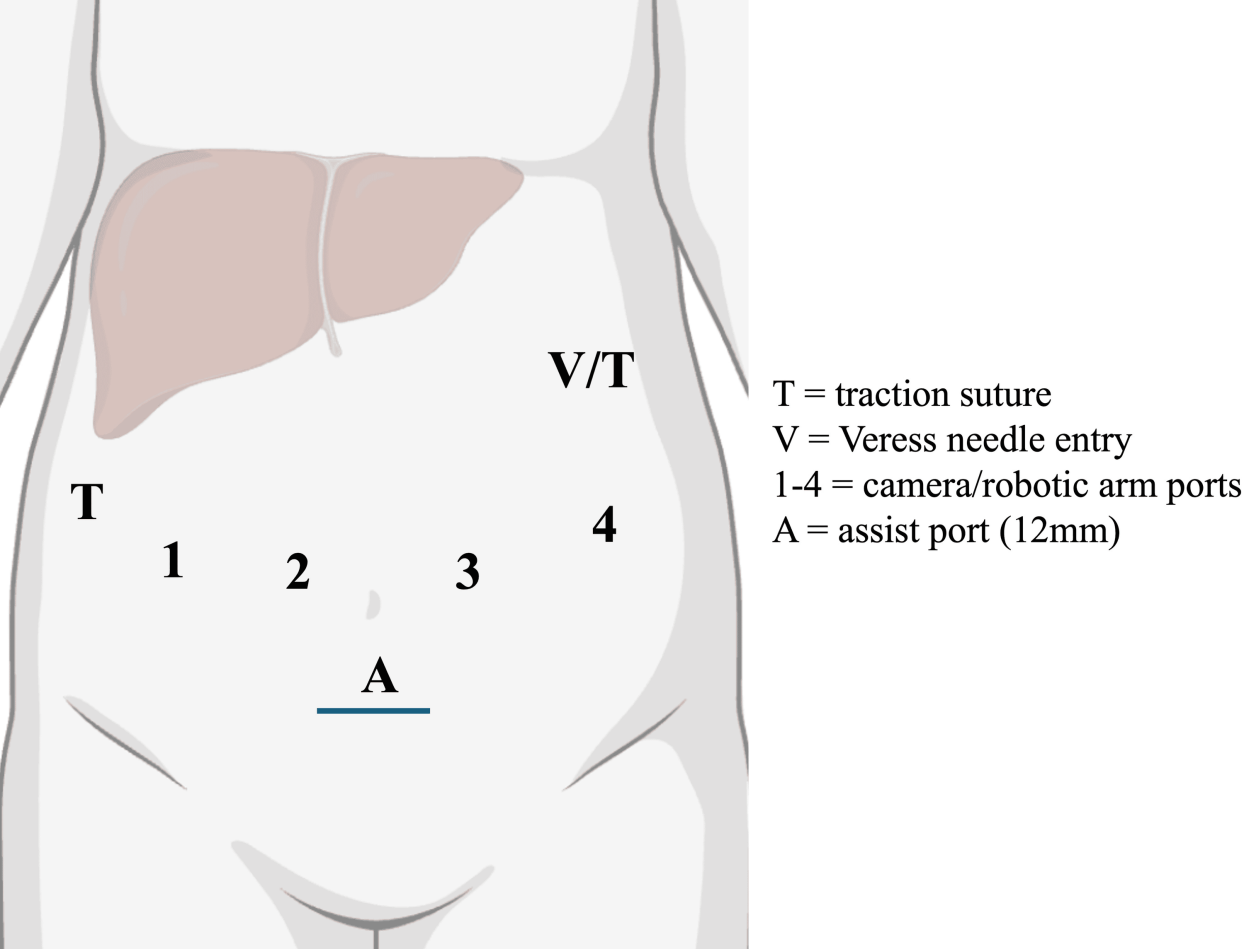
Port placement for robotic-assisted right hepatectomy.

The patient was brought to the operating room for robotic-assisted right hepatectomy and cholecystectomy. The abdomen was accessed in the left upper quadrant using a Veress needle, pneumoperitoneum was established, and four robotic ports were placed across the mid-abdomen with an additional 12-mm assistant port (Figure 2). Initial exploration demonstrated several small pale nodules on the liver surface which were biopsied and found to be benign biliary hamartomas. No additional intrahepatic or extrahepatic lesions were identified.

The operation began with division of the ligamentum teres and falciform ligament. Dissection was carried along the anterior surface of the hepatic veins, and a plane was developed between the inferior vena cava and the left and middle hepatic veins. The ligamentum venosum was opened, and the caudate lobe and caudate process were partially mobilized off the inferior vena cava. A tunnel was then developed anterior to the inferior vena cava and posterior to the left and middle hepatic veins (Video 1). A vessel loop was passed behind and around the left and middle hepatic veins in a double-loop fashion and secured for later venous control during parenchymal transection (Video 2). The robotic platform was particularly useful during this maneuver because its three-dimensional visualization and wristed instrumentation facilitated precise dissection in the narrow plane between the inferior vena cava and the left-middle hepatic venous confluence. These features enabled safe development of the retrohepatic tunnel and accurate placement of the vessel loop around the left-middle hepatic venous confluence without vascular injury.

**Video 1:** Development of a tunnel between inferior vena cava and the hepatic veins to enable passage of a vessel loop for extrahepatic outflow control. This step demonstrates the robotic platform's ability to perform precise maneuvers in a confined anatomical space, a critical prerequisite for achieving secure vascular control prior to parenchymal transection.

**Video 2:** Vessel loop encirclement of the left and middle hepatic veins to control venous outflow. The vessel loop allows the surgeon to tighten the loop and temporarily occlude the veins to significantly reduce venous back-bleeding during parenchymal transection.

Attention was then turned to the gallbladder and hilar dissection. The cystic artery and cystic duct were dissected, clipped, ligated, and divided, and the gallbladder was removed from the gallbladder fossa. The cystic duct stump was used to elevate the bile duct and facilitate exposure of the right hepatic artery, which was circumferentially dissected, ligated, clipped, and divided. The right portal vein was then meticulously dissected, and several inferior branches were identified and divided with a vessel sealer. The vein was then encircled with a 2-0 silk tie and temporarily occluded with a bulldog clamp. After temporary occlusion, ICG was administered, but persistent perfusion of the right liver suggested an additional arterial source. The right portal vein was divided after confirming the left portal vein anatomy. Further hilar dissection identified an anterior sectoral branch of the right hepatic artery, which was controlled and divided. After control of this additional arterial branch, ICG assessment demonstrated clear demarcation of the right liver (Video 3).

**Video 3:** Intraoperative indocyanine green assessment. Intraoperative indocyanine green assessment shows clear demarcation of right liver after temporary occlusion of right portal vein and division of both right hepatic artery and anterior sectoral branch of right hepatic artery.

Intraoperative ultrasound was used to confirm the location of the segment 7 tumor, exclude additional lesions, and map the course of the left hepatic vein (Video 4). The porta hepatis was then encircled with a 14-Fr catheter to create a Huang loop. If bleeding is encountered during parenchymal transection, the Huang loop can be used for intermittent Pringle maneuver to obtain inflow control along with simultaneous vessel loop tightening for venous outflow control (Video 5). Although the Pringle maneuver can be applied for up to 10-15 minutes continuously, the vessel loop occlusion of the left-middle hepatic venous confluence is usually only applied briefly and selectively during high-risk portions of parenchymal transection or when venous back-bleeding is encountered. The right liver was mobilized from the retroperitoneum with careful dissection along the inferior vena cava. Multiple retrohepatic venous branches were controlled using a vessel sealer, clips, and ties. A tunnel was developed behind the liver between the middle and right hepatic veins, and umbilical tape was passed through this tunnel to assist with mobilization and exposure.

**Video 4:** Intraoperative ultrasound for mapping the course of the left hepatic vein. Real-time ultrasonographic guidance confirms the anatomical relationship between the left hepatic vein and the planned transection plane, ensuring accurate and safe parenchymal division.

**Video 5:** Encirclement of the porta hepatis with a 14-Fr catheter for a Huang loop Pringle maneuver. Combined with extrahepatic outflow control of the hepatic veins, securing inflow using a Pringle maneuver provides comprehensive vascular control of the liver, enabling parenchymal transection with minimal blood loss.

Parenchymal transection was then performed under intermittent Pringle control using a saline-cooled double bipolar technique (Video 6). Larger venous branches were controlled with the vessel sealer, clips, and stapler loads as needed. Branches of the middle hepatic vein draining segments 5 and 8 were controlled and divided. The right bile duct was encircled and divided with a stapler. The dissection was continued down to the anterior surface of the inferior vena cava between the right and middle hepatic veins. The right hepatic vein was circumferentially dissected from a medial approach, encircled with a 2-0 silk tie, and divided with a 30 mm blue load stapler. The remaining retroperitoneal attachments were divided, and the right liver was completely removed.

**Video 6:** Intraoperative management of bleeding during parenchymal transection. Hemostatic mesh was initially applied to the bleeding site, after which the pre-placed vessel loop around the left and middle hepatic veins was tightened to reduce venous back-bleeding and achieve hemorrhage control. This sequence illustrates the critical safety advantage of obtaining extrahepatic outflow control prior to transection, enabling a rapid response to intraoperative bleeding.

Hemostasis was confirmed along the transection surface, and no bile leak was observed. Intraoperative ultrasound demonstrated preserved inflow and outflow to the future liver remnant, including segment 4 and the left and middle hepatic vein branches. The gallbladder and right liver were placed into separate specimen retrieval bags and removed through the enlarged infraumbilical assistant port site. Estimated blood loss was 100 mL, and no blood product transfusion was required. The patient was extubated and transferred to the surgical intensive care unit in stable condition for postoperative monitoring.

Her inpatient postoperative course was uncomplicated. Her diet was advanced as tolerated, and pain was initially controlled with patient-controlled analgesia and later transitioned to an oral regimen. Her Foley catheter was removed on postoperative day 2, and she had return of bowel function the same day. She remained afebrile and hemodynamically stable throughout the admission. She was discharged home in good condition on postoperative day 4. She was readmitted two weeks later with fever and chills and found to have a small nonbilious perihepatic collection, which was treated with antibiotics and drainage with prompt recovery. Culture and Gram stain of the fluid obtained from drain placement resulted in no growth on culture and only 4+ white blood cells with no organisms seen. After discharge, she recovered without difficulty or complications and was started on adjuvant chemotherapy.

Pathology of the resected right hepatectomy specimen showed a 4.5 cm metastatic adenocarcinoma of colon primary with small areas of residual viable tumor, approximately 15% of tumor volume, and negative resection margins. Over the next six months postoperatively, she completed eight cycles of adjuvant FOLFOX plus bevacizumab and was transitioned to active surveillance, with the most recent abdominal/pelvic CT scan at one year postoperatively showing no evidence of recurrent disease.

## Discussion

Robotic right hepatectomy remains one of the most technically demanding procedures in minimally invasive hepatobiliary surgery [5-7]. Although the robotic platform offers three-dimensional visualization and improved instrument articulation, these advantages do not eliminate the central operative challenge of controlling major hepatic vascular structures during parenchymal transection [8,9]. In right hepatectomy, bleeding from branches of the middle hepatic vein can be particularly difficult as the anatomical course can vary and may be encountered deep within the transection plane [10]. By obtaining early extrahepatic control of the left and middle hepatic veins before liver transection, this case report demonstrates reproducible intraoperative maneuvers which can help optimize safety and minimize bleeding during robotic-assisted right hepatectomy.

The key technical feature of this case was the development of a tunnel anterior to the inferior vena cava and posterior to the left and middle hepatic veins, allowing the left and middle hepatic veins to be encircled with a vessel loop in a double-loop fashion. This maneuver provided a controlled, temporary outflow occlusion during parenchymal transection. In combination with intermittent Pringle maneuver for inflow control, this approach allowed both inflow and outflow modulation during critical, high-risk portions of the parenchymal transection. This is particularly relevant in cases where the tumor involves the right hepatic vein or posterior right liver, as complete oncologic resection may require dissection close to key vascular structures including the inferior vena cava, middle hepatic vein branches, and major venous tributaries. In this patient, the segment 7 colorectal liver metastasis demonstrated persistent right hepatic vein involvement after neoadjuvant chemotherapy, making right hepatectomy necessary for complete resection. Despite this complexity, estimated blood loss was 100 mL, no blood transfusion was required, and no intraoperative bile leak was observed.

This case also highlights the value of utilizing multiple intraoperative tools and techniques, such as ultrasound and ICG assessment, to improve intraoperative decision-making during complex liver resection. Intraoperative ultrasound was used to confirm the tumor location, exclude additional lesions, and map the course of the middle hepatic vein - these were essential for defining the transection plane and anticipating the location of major venous branches. In addition, ICG assessment further contributed to operative safety by confirming right liver demarcation after inflow control. Notably, persistent right-sided perfusion after initial vascular control prompted additional hilar dissection and identification of an anterior sectoral right hepatic artery branch, which was then controlled before transection. This emphasizes how ICG assessment may be particularly useful in minimally invasive hepatectomy when vascular anatomy is variant or in cases where visual confirmation of adequate inflow control is uncertain.

The robotic platform is especially useful for complex hepatic surgeries which require extensive vascular dissection. For example, the enhanced visualization and improved instrument articulation can facilitate fine dissection around critical structures including the hepatic veins, inferior vena cava, and porta hepatis. However, these benefits must be balanced against the need for careful patient selection, advanced hepatobiliary expertise, and a low threshold for conversion if vascular control cannot be safely obtained. In our case, a minimally invasive approach was favored because of the patient’s significant cardiopulmonary comorbidities and the potential benefit of reducing the physiologic burden of an open operation. Her postoperative course was initially uncomplicated, and she was discharged on postoperative day 4; however, she was later readmitted with a small nonbilious perihepatic collection that resolved promptly with antibiotics and drainage. This postoperative event emphasizes that even when intraoperative vascular and biliary control are successful, complex liver resection still carries meaningful postoperative risk and requires close follow-up.

## Conclusions

This case demonstrates that robotic-assisted right hepatectomy with early extrahepatic control of the left and middle hepatic veins is feasible in a selected patient. By combining pre-transection outflow control, intermittent inflow occlusion, intraoperative ultrasound, and ICG perfusion assessment, this approach may help reduce the risk of uncontrolled venous bleeding during complex minimally invasive liver resection. Further experience is needed to determine whether this technique can be applied more broadly and whether it improves perioperative outcomes compared with conventional robotic right hepatectomy strategies.
